# Deep learning to distinguish Best vitelliform macular dystrophy (BVMD) from adult-onset vitelliform macular degeneration (AVMD)

**DOI:** 10.1038/s41598-022-16980-z

**Published:** 2022-07-26

**Authors:** Emanuele Crincoli, Zhanlin Zhao, Giuseppe Querques, Riccardo Sacconi, Matteo Maria Carlà, Federico Giannuzzi, Silvia Ferrara, Nicolò Ribarich, Gaia L’Abbate, Stanislao Rizzo, Eric H. Souied, Alexandra Miere

**Affiliations:** 1 Department of Ophthalmology, Centre Hospitalier Intercommunal de Créteil, 40, avenue de Verdun, 94100 Créteil, France; 2 Ophthalmology Unit, Fondazione Policlinico Universitario A. Gemelli IRCCS, Largo Agostino Gemelli 8, 00166 Rome, Italy; 3 Catholic University of “Sacro Cuore”, Largo Francesco Vito 1, 00166 Rome, Italy; 4 Department of Ophthalmology University Vita-Salute IRCCS San Raffaele Scientific Institute, Via Olgettina, 60, 20132 Milan, Italy; 5 Ethics Committee of the Federation France Macula, 2018-27, 40 Av. de Verdun, 94010 Créteil, France

**Keywords:** Data processing, Diagnostic markers

## Abstract

Initial stages of Best vitelliform macular dystrophy (BVMD) and adult vitelliform macular dystrophy (AVMD) harbor similar blue autofluorescence (BAF) and optical coherence tomography (OCT) features. Nevertheless, BVMD is characterized by a worse final stage visual acuity (VA) and an earlier onset of critical VA loss. Currently, differential diagnosis requires an invasive and time-consuming process including genetic testing, electrooculography (EOG), full field electroretinogram (ERG), and visual field testing. The aim of our study was to automatically classify OCT and BAF images from stage II BVMD and AVMD eyes using a deep learning algorithm and to identify an image processing method to facilitate human-based clinical diagnosis based on non-invasive tests like BAF and OCT without the use of machine-learning technology. After the application of a customized image processing method, OCT images were characterized by a dark appearance of the vitelliform deposit in the case of BVMD and a lighter inhomogeneous appearance in the case of AVMD. By contrast, a customized method for processing of BAF images revealed that BVMD and AVMD were characterized respectively by the presence or absence of a hypo-autofluorescent region of retina encircling the central hyperautofluorescent foveal lesion. The human-based evaluation of both BAF and OCT images showed significantly higher correspondence to ground truth reference when performed on processed images. The deep learning classifiers based on BAF and OCT images showed around 90% accuracy of classification with both processed and unprocessed images, which was significantly higher than human performance on both processed and unprocessed images. The ability to differentiate between the two entities without recurring to invasive and expensive tests may offer a valuable clinical tool in the management of the two diseases.

## Introduction

Bestrophinopathies are a group of retinal diseases whose common characteristic is the presence of a mutation in *BEST1* gene in the affected patient. Best vitelliform macular dystrophy (BVMD), whose inheritability was clinically identified in 1905^1^, was the first of the group to be linked to *BEST1* mutations^2,3^. Since then, mutations in *BEST1* have been found in association with at least four additional clinically distinct retinal degenerative diseases, namely autosomal recessive bestrophinopathy, adult-onset vitelliform macular dystrophy (AVMD), autosomal dominant vitreoretinochoroidopathy, and retinitis pigmentosa^4,5^. *BEST1* gene encodes for bestrophin1, a basolateral membrane protein specific to retinal pigmented epithelium (RPE) cells, meaning that no expression can be found in the neurosensory retina, ciliary body, iris, cornea, or lens^6–8^. The protein functions as an anion channel, playing an important role in the determination of transepithelial electrical potential and regulation of intracellular Ca^2+^ signaling^9–12^. As a consequence, mutation of *BEST1* has been demonstrated to alter metabolism and cellular homeostasis through induction of protein mis-trafficking^13^, defects in BEST1 channel oligomerization^14^ and activity^15^ and impairment in intracellular calcium signaling^16^. BVMD is inherited in an autosomal dominant fashion and is mostly associated to missense mutations in *BEST1* gene causing impairment in channel activity^7^. As with many autosomal dominant diseases, however, there is variability in both expression and age of disease presentation^17,18^. By contrast, AVMD is a usually sporadic disease that has been associated with mutations both in *BEST1* and *peripherin/RDS* (*PRPH2*), even though the majority of cases appear to be idiopathic (negative to genetic testing)^5^. The incidence of AVMD is believed to be 3 times higher than BVMD^19^, although the lack of genetic testing highly impairs accuracy of the diagnosis and the ability to detect genetic characteristics of the disease. This complex genetic frame accounts for the fact that genetic testing alone is not sufficient for a correct definition of the two entities and should be supported by functional and morphological exams. In fact, the current gold standard for the diagnosis of these two clinical entities requires a comprehensive evaluation including genetic testing, family and personal medical history, fundoscopy, optical coherence tomography (OCT) and blue autofluorescence (BAF) images, electrooculography (EOG), full field electroretinogram (ERG), visual acuity and visual field testing^17^.

AVMD and early stages BVMD harbor similar BAF and OCT features which make it difficult for the human eye to distinguish between the two based on these two routinary, cost-effective and minimally invasive exams. Nevertheless, the functional prognosis in highly different in most cases, BVMD being characterized by a worse final stage VA and an earlier onset of critical VA loss. In fact, the final stage of classical BVMD is associated with severe vision loss and can be characterized by either retinal atrophy/hypopigmentation (IVa), or scarring with fibrous hyperpigmented tissue in the macula(IVb) or choroidal neovascularization beneath and around the gliotic macular scar (IVc)^18^. In this perspective, the ability to differentiate between the two entities without recurring to invasive and expensive tests may offer a valuable clinical tool in the management of the two diseases. The aim of our study is therefore to automatically classify OCT and BAF images from stage II BVMD and AVMD eyes using a deep learning algorithm to facilitate clinical diagnosis. Moreover, we aim to identify an image processing method to facilitate human-based clinical diagnosis based on non-invasive tests like BAF and OCT without the use of machine-learning technology.

## Materials and methods

This multicentric retrospective study analyzed patients referring for BVMD or AVMD to the Department of Ophthalmology of the University Paris Est Creteil, the Department of Ophthalmology of the Fondazione Policlinico Universitario Agostino Gemelli and the Department of Ophthalmology of IRCSS San Raffaele Milan from September 2018 to December 2021.

The ground truth reference for the diagnosis of BVMD and AVMD was set from two experts in the field and was based on comprehensive evaluation of genetic testing, family and personal medical history, fundoscopy, OCT and BAF images, EOG, full-field ERG, visual acuity and visual field testing^18^. In fact, the combination of multimodal imaging, genetic testing results and functional exams is currently still considered as the most accurate method for BVMD diagnosis^20^. In particular, only patients with available genetic testing results and EOG results were considered for the analysis. Moreover, only patients in stage II BVMD according to Mohler and Fine classification were included and only unifocal variants with macular involvement were considered^21^. BVMD diagnosis was performed by the two expert graders in a blinded fashion and was confirmed only in case of agreement between the two. In case of disagreement, controversies were solved by referring to a third expert grader. As concerns genetic testing, the following mutations in *BEST1 *(VMD2) gene were considered as suggestive of BVMD, due to a well-documented association in literature^17^: in-frame deletion Ile295del, 1574delCA frameshift mutation, *BEST1* gene missense mutations. Genetic characteristics suggestive of AVMD were: *PRPH2* mutation and negative genetic testing^17,22^. Exclusion criteria were the concomitant presence of other retinal pathologies, retinal atrophy, previous vitreoretinal surgery, media opacity, axial length > 26 mm or < 22.5 mm.

For each patient all available macula-centered enface BAF retinal images and OCT B-scan acquisitions were collected. More than one image for both OCT and BAF was included for each patient, since images from different time points were considered for the same subject. All images had been acquired using Spectralis HRA + OCT (Heidelberg Eye Explorer, Version 1.10.4.0, Heidelberg Engineering, Heidelberg, Germany). Included images had a minimum quality score of 8. BAF images had to be high-resolution (1536 × 1536 pixels), 30 × 30 degree-field-of-view images centered on the fovea with a minimum average of 30 frames. OCT images were high resolution horizontal B-scan images of 200 × 200 mm dimensions centered on the fovea. All images were deidentified, and all personal data (e.g., patient name, birth date, and study date) were removed.

Two different human graders (E.C, Z.Z.) then classified randomly presented B-scan OCT unprocessed images as belonging to patients affected by either BVMD or AVMD. The same procedure was repeated presenting enface BAF images of the study participants. Typical ocular fundus presentation of AVMD is that of a vitelliform-like lesion of about 500 to 700 microns in size associated with only a minimal or mild amount of visual loss. The dome-shaped lesion located between the RPE and the photoreceptor layers is characterized by hyperautofluorescence on BAF and by hyperreflectivity on OCT examination^23^. Similarly, BAF of stage 2 BVMD patients usually shows either diffuse or patchy hyperautofluorescence of the vitelliform lesion^24^, with subsequent complete hypoautofluorescent at later atrophic stages of the disease^25^. Likewise, OCT imaging at vitelliform stage of BVMD features a subretinal dome-shaped lesion filled with hyperreflective material whose aspect is extremely close to that of AVMD lesions. The lesion subsequently evolves with a decrease and scrambling of the hyperreflective material and concomitant loss of photoreceptors ending with RPE atrophy^18^.

### Image processing

**Figure 1:**
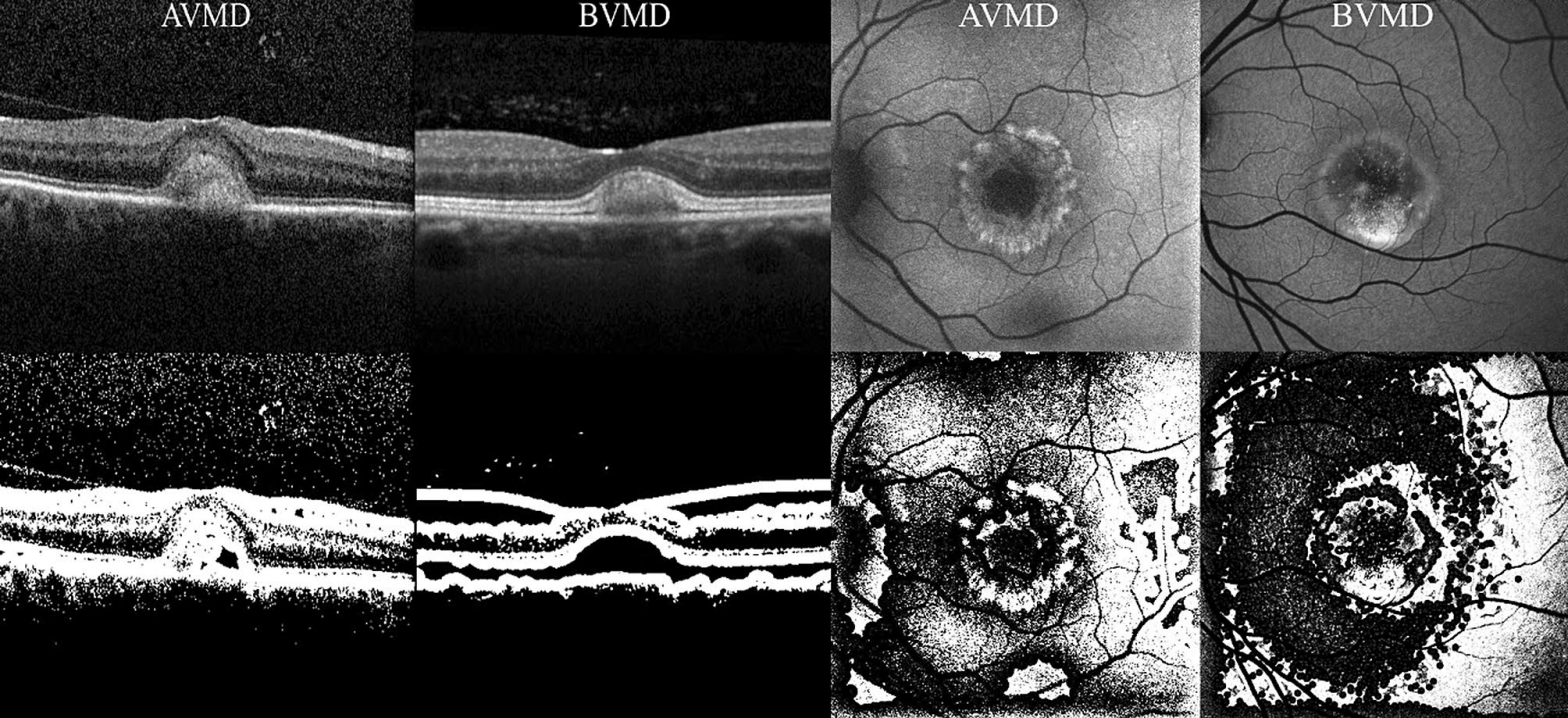
OCT (left quadrant) and BAF (right quadrant) images before (first row) and after (second row) image processing. *BAF* blue autofluorescence, *OCT* optical coherence tomography.

Image processing was performed using an open-source image processing software (ImageJ, NIH, Bethesda, MD). All images were converted in 8-bit mono-dimensional images. Both OCT B-scan and BAF images were than elaborated using an auto local thresholding method for binarization. Local thresholding is a group of procedures in which the threshold for each pixel is computed in relation to the image characteristics within a window of radius r (in pixel units) of pixels around it. Pixels whose luminance is above the locally calculated threshold are always shown as white (255 intensity). In particular, BAF images were elaborated with a contrast-based local thresholding method, which sets the pixel value to either white (255) or black (0) depending on whether its current value is closest to the local maximum or minimum respectively (threshold in this case is represented by the median of the local luminance) (see Fig. 1)^26^. By contrast, OCT images were modified using a mean-based method, that selects the threshold for binarization as the mean of the local greyscale distribution (threshold in this case is represented by the mean of the local luminance) (see Fig. 1)^26^. For both BAF and OCT images a kernel radius of 8 was applied. The same blinded human graders that performed binary image classification (BVMD or AVMD) of unprocessed images were then asked to perform twice again the same task, this time based on B-scan OCT and BAF processed images, respectively.

### Deep learning classifier

MatLab software (Mathworks, Natick, MA) deep learning toolbox was used as a framework for the deep learning process. Images were classified using Inception-ResNet-v2 convolutional neural network (CNN)^27^. Transfer learning using the ImageNet dataset (http://www.image-net.org/) was performed. Images from the Department of Ophthalmology of the University Paris Est Creteil were used for training (85% of the set of images) and validation (15% of the set of images). Testing was performed using images of external centers (Department of Ophthalmology of Fondazione Policlinico Universitario Agostino Gemelli and Department of Ophthalmology of IRCSS San Raffaele Milan). To fit our task, we reduced the number of output neurons in the last fully connected layer to two. The images were automatically normalized by the CNN according to the default dimensions of the classifier (229 × 229 pixels). Augmentation techniques such as rotation (from − 20° to + 20°) and horizontal translation (from − 2.00 to + 2.00) were used to increase the original dataset. Mini batch size was set to 32. Additional regularization strategies included weight constrain and introduction of drop out layers. Gradient-weighted Class Activation Mapping (Grad-CAM)^28^ was used as a visualization method, allowing detection of discriminative regions of the images that the model relied on to perform classification.

### Statistical analysis

Statistical analysis was performed using SPSS v.26 (IBM SPSS Statistics). Cohen’s kappa analysis was used to assess agreement between graders in human-based classification of both unprocessed and processed BAF and OCT images. Accuracy, sensitivity, and specificity of each method either for human-based image processing classification or CNN-based classification were assessed using confusion matrices. Area under the receiver operating characteristics (AUROC) curves was determined to evaluate the model performances. AUROC from the different methods were compared using DeLong test. A *p* value < 0.05 was considered as statistically significant.

### Ethics approval

All procedures performed in studies involving human participants were in accordance with the ethical standards of the institutional and/or national research committee and with the 1964 Helsinki declaration and its later amendments or comparable ethical standards.The study was approved by the Ethics Committee of the Federation France Macula 2018–29.

### Consent to participate

Informed consent was obtained from all individual participants included in the study.

## Results

A total of 118 BVMD eyes (355 SD-OCT images and 325 BAF images) and 96 AVMD eyes (287 SD-OCT images and 208 BAF images) were included. The number of analyzed images was 642 for SD-OCT (355 from BVMD eyes and 287 from AVMD eyes) and 533 images for BAF (325 from BVMD eyes and 208 from AVMD eyes). Mean age of the population was 47.8 ± 9.2 years for BVMD and 51.9 ± 8.5 years for AVMD respectively (*p* = 0.645). Among BVMD patients, 68 (57.6%) were of male sex, while AVMD showed a male sex prevalence of 51.0% (49 patients) (*p* = 0.371). Mean best corrected visual acuity (BCVA) at the time of image acquisition was 80.5 ETDRS letters in BVMD and 78.7 letters in AVMD group (*p* = 0.828). Only two patients in the testing set of AVMD group showed a mutation in *BEST1* gene (Arg47His), the others being positive for mutation in *PRPH2* or negative to genetic testing. All patients in the BVMD group tested positively for a disease-causing mutation in *BEST1* gene.

### Deep learning versus human classification using BAF images

**Figure 2:**
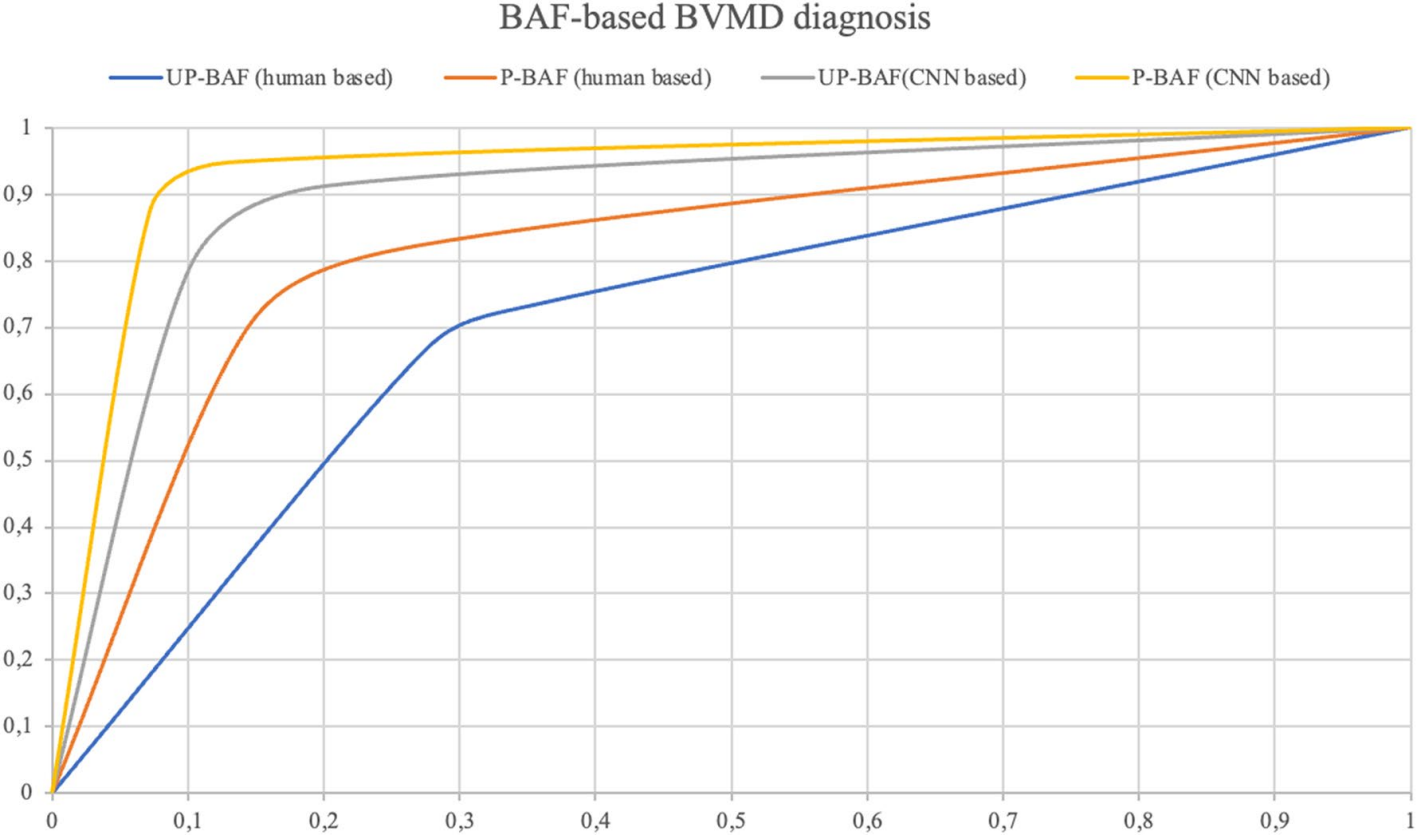
ROC curves illustrating accuracy of the 4 classification methods on BAF images. *BAF* blue autofluorescence, *CNN* convolutional neural network, *P-BAF* processed BAF images, *UP-BAF* unprocessed BAF images.

Both BAF deep learning classifiers were trained on 346, validated on 61 and tested on 126 images. Among test BAF dataset images, 69 belonged to BVMD eyes and 57 to AVMD eyes. Both human-based methods (diagnosis attribution on either unprocessed or processed images without the use of a CNN) were performed on the same set of images used for testing in CNN-based classifications to increase homogeneity of the accuracy evaluation. Human-based classification of BAF unprocessed images was characterized by a moderate agreement between graders (k = 0.571, CI 0.543–0.582) and reveled a sensitivity of 65.2%, specificity of 66.7%, a positive predictive value (PPV) of 70.3% and negative predictive value (NPV) of 61.3% in BVMD diagnosis (AUROC = 0.614, CI 0.557–0.672) (Table 1, Fig. 2). BAF processed images of BVMD and AVMD were characterized respectively by the presence or absence of a hypoautofluorescent region of retina encircling the central hyperautofluorescent foveal lesion (see Fig. 1). In fact, this characteristic was typically associated with BAF images of BVMD, and its detection was associated with a good agreement between graders (k = 0.675, CI 0.669–0.682) (see Fig. 1). Diagnosis attribution based on this assumption led to a sensitivity of 79.9% a specificity of 80.7%, a PPV of 83.1% and a NPV of 75.4% in classification of the two entities (AUROC = 0.785, CI 0.768–0.810) (Table 1, Fig. 2). The deep learning classifier trained with unprocessed BAF images was characterized by a sensitivity of 86.9%, a specificity of 87.7%, a PPV of 89.5% and a NPV of 84.7% in detection of BVMD (AUROC = 0.861, CI 0.843–0.879) (Table 1, Fig. 2). Lastly, the deep learning classifier trained with processed BAF images led to an AUROC of 0.880 (CI 0.862–0.896) (sensitivity = 89.9%, specificity = 89.5%, PPV = 91.2%, NPV = 87.9%) (Fig. 2).

**Table 1:** Classification matrices describing the performances on BAF images of human-based methods on both unprocessed (UP) and processed (P) images and CNN-based methods on both UP and P images.

BAF images		UP human-based	
		Positive	Negative
Ground truth	Positive	45	24
	Negative	19	38

		P human-based	
		Positive	Negative
Ground truth	Positive	54	15
	Negative	11	46

		UP CNN-based	
		Positive	Negative
Ground truth	Positive	60	9
	Negative	7	50

		P CNN-based	
		Positive	Negative
Ground truth	Positive	62	7
	Negative	6	51

*BAF *blue autofluorescence, *CNN *convolutional neural network.

### Deep learning versus human classification using OCT images

**Figure 3:**
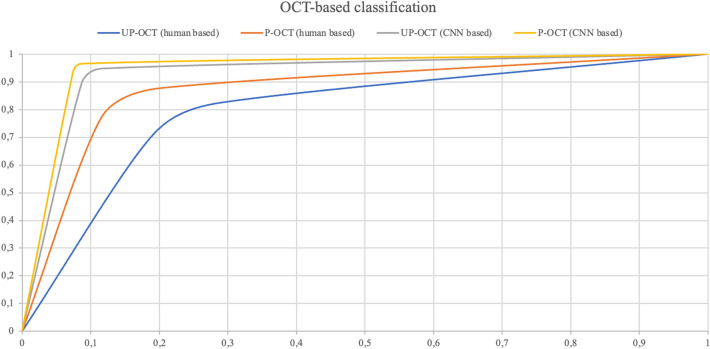
ROC curves illustrating accuracy of the 4 classification methods on OCT images. *CNN* convolutional neural network; *OCT* optical coherence tomography, *P-OCT* processed OCT images, *UP-OCT* unprocessed OCT images.

**Figure 4:**
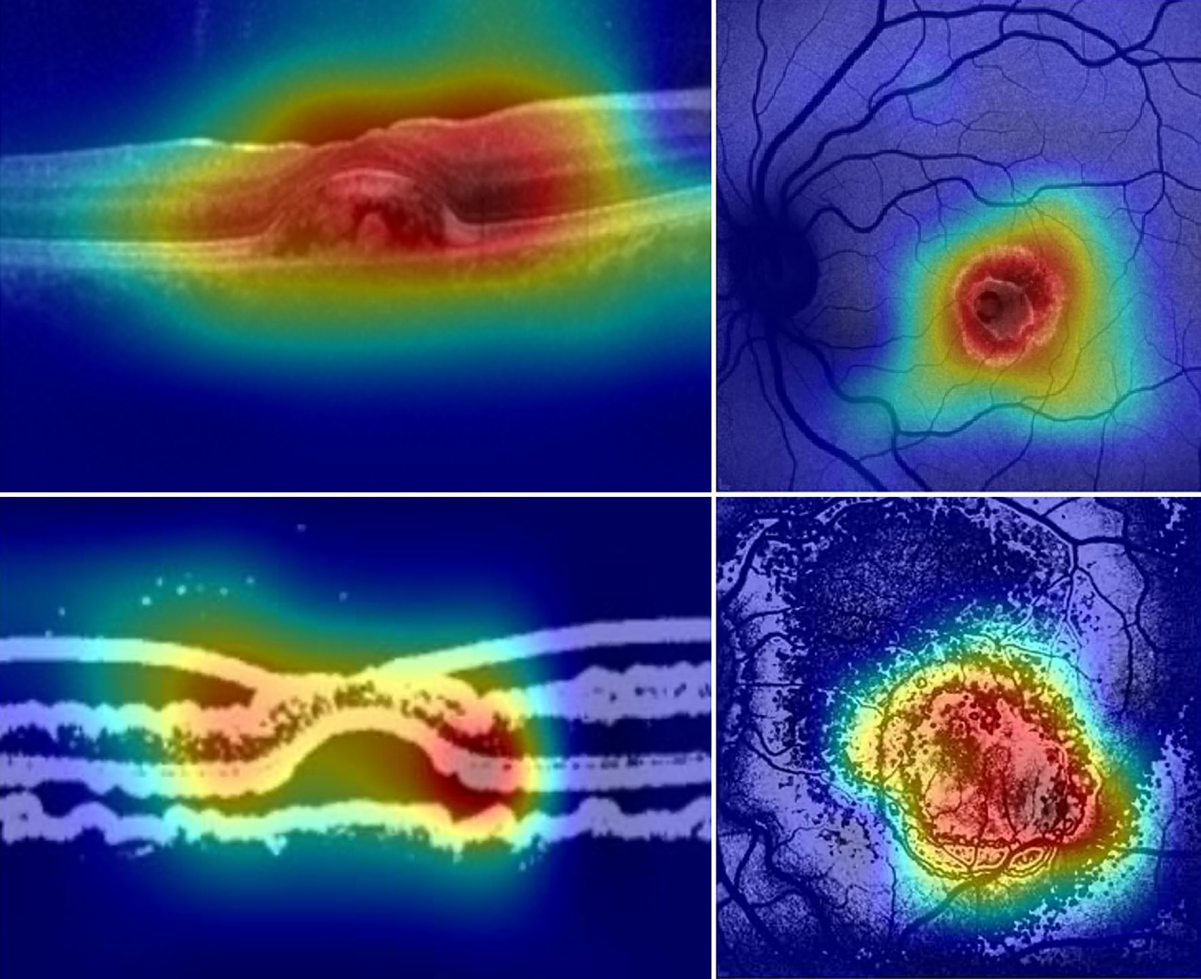
GradCAM output highlighting relevant features for each of the 4 deep learning classifiers. Upper left: deep learning classifier for unprocessed OCT images; Upper right: deep learning classifier for unprocessed BAF images; Lower left: deep learning classifier for OCT processed images; Lower right: deep learning classifier for BAF processed images. *BAF* blue autofluorescence, *OCT* optical coherence tomography.

Among the 631 available OCT images, both the unprocessed-images-based and the processed-images-based deep learning classifier were trained with 429, validated with 76 and tested with 126 images (69 BVMD images and 57 AVMD images). The performances of the human-based methods (considering either processed or unprocessed images) were evaluated on the same set of 126 images. Human-based evaluation showed a sensitivity of 66.7%, a specificity of 68.4%, a PPV of 71.9% and a NPV of 62.9% (AUROC 0.662, CI 0.657–0.684), with a moderate agreement between graders (k = 0.581, CI 0.563–0.596). OCT processed images were characterized by hyporeflectivity of the vitelliform deposit in the case of BVMD and hyperreflectivity with high internal inhomogeneity in the case of AVMD (see Fig. 1). Diagnosis attribution based on processed images was characterized by a good inter-grader agreement (k = 0.682, CI 0.645–0.699), a sensitivity of 75.4%, a specificity of 77.2%, a PPV of 80.6% and a NPV of 72.1% (AUROC = 0.741, CI 0.718–0.757). The deep learning method based on unprocessed images showed a sensitivity of 88.4%, a specificity of 89.5%, a PPV of 91.0% and a NPV of 86.4% (AUROC = 0.867, CI 0.853–0.881). Lastly, the deep learning method based on processed images was characterized by a sensitivity of 91.3%, a specificity of 91.2%, a PPV of 92.6% and a NPV of 89.6% (AUROC = 0.893, CI 0.882–0.911) (see Table 2, Fig. 3). Relevant features for classification were highlighted using gradCAM method on each of the four deep learning classifiers (see Fig. 4).

**Table 2:** Classification matrices describing the performances on OCT images of human-based methods on both unprocessed (UP) and processed (P) images and CNN-based methods on both UP and P images.

OCT images		UP human-based	
		Positive	Negative
Ground truth	Positive	46	23
	Negative	18	39

		P human-based	
		Positive	Negative
Ground truth	Positive	52	17
	Negative	13	44

		UP CNN-based	
		Positive	Negative
Ground truth	Positive	61	8
	Negative	6	51

		P CNN-based	
		Positive	Negative
Ground truth	Positive	63	6
	Negative	5	52

*CNN *convolutional neural network, *OCT *optical coherence tomography.

### Performance comparison between BAF and OCT with unprocessed versus processed images

Comparison of AUROCs from the 4 different methods for classification of BAF images showed a statistically significant difference (*p* < 0.001). Specifically, according to post-hoc analysis, human-based classification of unprocessed images (UPhb) was significantly less performing than both human-based classification of processed images (Phb) (*p* = 0.019), CNN classification of unprocessed images (UP CNN) (*p* < 0.001) and CNN classification of processed images (P CNN) (*p* < 0.001). Moreover, Phb method performed worse than both UP CNN (*p* = 0.009) and P CNN (*p* = 0.005) while no significant difference was found between UP CNN and P CNN methods (*p* = 0.441). Similarly, AUROCs deriving from the 4 methods of classification of OCT images showed statistically significant differences (*p* < 0.001). In particular, UPhb method showed significantly lower accuracy compared to both Phb (*p* = 0.041), UP CNN (*p* < 0.001) and P CNN (*p* < 0.001). In addition, UP CNN and P CNN didn’t differ significantly between each other (*p* = 0.518) but were both significantly more accurate than Phb (*p* = 0.008 and *p* = 0.006 respectively) (see Table 3). Human-based differential diagnosis based on unprocessed OCT images was significantly more accurate than the one performed on unprocessed BAF images (*p* = 0.031). Moreover, human interpretation of processed BAF images performed better than the one on processed OCT images (*p* = 0.025). Lastly there were no differences in the performance of the CNN both based on unprocessed BAF and OCT images (*p* = 0.652) and on processed BAF and OCT images (*p* = 0.790) (see Table 4).

**Table 3:** Comparison of performances (AUROCs) of the 4 different methods on BAF images (first row) and OCT images (second row).

	UP hb	P hb	UP CNN	P CNN	*p*
BAF images	0.614 (CI 0.557–0.672)	0.785(CI 0.768–0.810)	0.861(CI 0.843–0.879)	0.880 (CI 0.862–0.896)	< 0.001
OCT images	0.662 (CI 0.657–0.684)	0.741 (CI 0.718–0.757)	0.867(CI 0.853–0.881)	0.893(CI 0.882–0.911)	< 0.001

The analysis responds to the question “which method performed better in differential diagnosis based on BAF/OCT images?”. *BAF* blue autofluorescence, *CNN* convolutional neural network, *OCT* optical coherence tomography, *P CNN* CNN classification of unprocessed images, *P hb* human-based classification of processed images, *UP CNN* CNN classification of unprocessed images, *UP hb* human-based classification of unprocessed images.

**Table 4:** Comparison of classification accuracy using either BAF or OCT images for each of the 4 described methods.

	BAF images	OCT images	*p*
UP hb	0.614 (CI 0.557–0.672)	0.662 (CI 0.657–0.684)	0.031
P hb	0.785 (CI 0.768–0.810)	0.741 (CI 0.718–0.757)	0.025
UP CNN	0.861 (CI 0.843–0.879)	0.867 (CI 0.853–0.881)	0.652
P CNN	0.880 (CI 0.862–0.896)	0.893 (CI 0.882–0.911)	0.790

The analysis responds to the question “Does UPhb/Phb/UP CNN/P CNN perform better when applied on BAF or OCT images?”. *BAF* blue autofluorescence, *CNN* convolutional neural network, *OCT* optical coherence tomography, *P CNN* CNN classification of unprocessed images, *P hb* human-based classification of processed images, *UP CNN* CNN classification of unprocessed images, *UP hb* human-based classification of unprocessed images.

## Discussion

Deep learning has been increasingly applied in inherited retinal diseases during the last years, challengers being the small datasets available due to the rarity of the disease. Miere et al.^29^ recently published the results of a BAF images based deep learning classifier trained for recognition of BVMD, Stargardt disease and retinitis pigmentosa and showing particularly good performances in the detection of retinitis pigmentosa images. We propose the use of deep learning for single-exam based differential diagnosis between vitelliform stage of BVMD and AVMD as a method that could facilitate screening and avoid the use of multiple invasive tests while efficiently distinguishing between two entities with very different prognostic implications for both the patient and his/her family. In fact, due to the high similarity between BVMD and AVMD acquisitions deriving from commonly performed imaging techniques, human-based definitive diagnosis is generally established taking into account patient’s age, genealogy and symptoms, but also functional testing (i.e. EOG) and molecular genetic testing^7^. Accurate diagnosis thus often requires the application of invasive, time-consuming and expensive tests, which may also pose ethical issues bringing to the attention unwanted information (such as in the case of genetic testing). Moreover, some of the above-mentioned diagnostic elements may sometimes be misleading. For example, approximately up to 7–9% of patients harboring disease-causing *BEST1* mutations have normal vision and do not exhibit decreased visual acuity^30^ while others report only episodic or mild vision loss^5,18^. Secondarily, age, genealogy and severity of symptoms may be misleading in the setting of mild or late onset variants or poor penetrance hereditary cases. Lastly, even though EOG anomalies such as the fall of LP/DT ratio below 1.55 have been found in all stages of BVMD, some patients show absence of abnormalities throughout the whole course of the disease^31,32^. In addition, EOG is an ancillary method that is rarely used in clinical practice, also due to the fact that its correct acquisition and interpretation require particular expertise.

In this context, an increase in reliability of the differential diagnosis performed with the use of other more common and less invasive methods such as BAF and OCT is particularly profitable. In our study, the best performance in classifying the two entities was obtained by the deep learning systems both in the case of BAF and OCT images. The CNN showed very high performances in analyzing both processed and unprocessed images. Moreover, both processed images-based CNN and unprocessed images-based CNN classifications showed equal performance (no significant differences) with either BAF or OCT images (see Table 4). Specifically, the classifier based on BAF images showed 86.9% sensitivity in the analysis of unprocessed images and 89.9% sensitivity in the analysis of processed images. This particularly high performances are even more valuable since they were both significantly more accurate than human-based classification based on either processed or unprocessed images. Interestingly, even the 2 patients within the AVMD group that tested positively for *BEST1* mutation were correctly classified by both the BAF and the OCT based CNNs.

Image processing of BAF images facilitated the distinction between the two entities to human graders, leading to a significantly higher correspondence to ground truth diagnosis compared to human-based analysis of unprocessed images. Human-based distinction of BAF processed images was based on the presence of hypoautofluorescent halos of variable size surrounding BVMD lesions that were not present in unprocessed images (see Fig. 1). Given the fact that processed images were the result of a local contrast-based filtering, this elaboration might have enhanced subtle local parafoveal decrease in autofluorescence. This, in turn, might be reflecting a disfunction in RPE metabolism involving a much larger area of the posterior pole than the one visible at raw BVMD BAF images. By contrast, AVMD might be characterized by a much more localized type of involvement. The hyperautofluorescence of the central lesion in both BVMD and AVMD is allegedly due to the subretinal deposition of lipofuscin generated by RPE disfunction in the turn-out of the photoreceptors’ outer segments. The surrounding hypoautofluorescent areas evidenced by image processing in BVMD might highlight areas of lower activity of the RPE that still retain enough functionality to avoid lipofuscin accumulation^6^. CNN analysis of both processed and unprocessed OCT images showed very high adherence to ground truth classification (respectively 91.3% and 88.4% sensitivity). GradCAM visualization highlighted the outer retinal layers overlying the center and the borders of the vitelliform lesion as significant discriminative regions (see Fig. 4). Interestingly, Ferrara et al.^33^ pointed out the thinning of the outer nuclear layer at the margins of the lesion and the thinning of the photoreceptors complex at the top of the lesion as frequent characteristics in OCT images of BVMD at vitelliform stage. The human-based analysis of OCT images also showed significantly higher correspondence to ground truth reference when performed on processed images (75.4% sensitivity versus 66.7% in the analysis of unprocessed images). Processed OCT images of BVMD fovea showed a dark appearance in correspondence to the vitelliform lesion while AVMD lesions appeared dishomogeneous and prevalently white (see Fig. 1). It should be kept in mind that these differences are the result of a processing mechanism aimed at enhancing the relationship between adjacent pixels. In this case, the dark appearance of pixels within the lesion is the result of a luminance that is below the threshold represented by the median luminance value within a radius of 5. This could be the result of even small luminance differences between adjacent surfaces, that are in this specific case the deposit’s material and the RPE/photoreceptors. The dark appearance could in fact not be interpreted as hyporeflectivity of the lesion but as a relative difference between the material and the adjacent surface which is homogeneous within the deposit and is not present in the case of AVMD. When comparing the performance of human interpretation of BAF and OCT images, it appears that humans performed better in classifying OCT images than BAF images before the application of the image processing method (*p* = 0.025, see Table 4). By contrast, classification performance of human graders was better when evaluating processed BAF images compared to processed OCT images. To conclude, deep learning methods on both processed and unprocessed BAF and OCT images proved to be highly effective in the distinction between early stage BVMD and AVMD. The application of this method could avoid the use of extensive evaluation and the need for genetic testing. The fact that both groups were composed of working-age patients gives an added value to the potential information deriving from a correct early diagnosis. Lastly, the multicentric nature of the study and the availability of an external set of images to perform testing should be mentioned among the strengths of the article. By contrast, limitations include the retrospective nature of the analysis and the mild asymmetry of the samples.

Yet, to the best of our knowledge this is the first study providing automated distinction between stage II BVMD and AVMD using everyday-practice methods and introducing image processing techniques that could offer a novel insight into the imaging features of the two diseases.

## Data Availability

Data are available upon reasonable request to the corresponding author.
